# Case report: An illusive cortical venous infarction mimicking glioma hemorrhage

**DOI:** 10.3389/fnins.2022.1075885

**Published:** 2022-12-08

**Authors:** Dayun Feng, Le Zou, Huaizhou Qin, Qing Cai

**Affiliations:** ^1^Department of Neurosurgery, Tangdu Hospital, Air Force Medical University, Xi'an, China; ^2^Clinical Skills Training Center, Tangdu Hospital, Air Force Medical University, Xi'an, China

**Keywords:** cortical vein thrombosis, cortical vein infraction, glioma hemorrhage, magnetic resonance venography, computerized tomography

## Abstract

Cortical vein thrombosis (CVT) is a rare subtype of cerebral venous thrombosis. Because CVT is rare and its clinical and imaging findings are atypical, the misdiagnosis of CVT may be extremely high. We report a case of cortical venous infarction (CVI) secondary to CVT. Due to the atypical symptoms, we were perplexed about confirming the diagnosis between CVI and glioma hemorrhage. Eventually, CVT was confirmed by pathology combined with imaging.

## Case report

**Figure 1:**
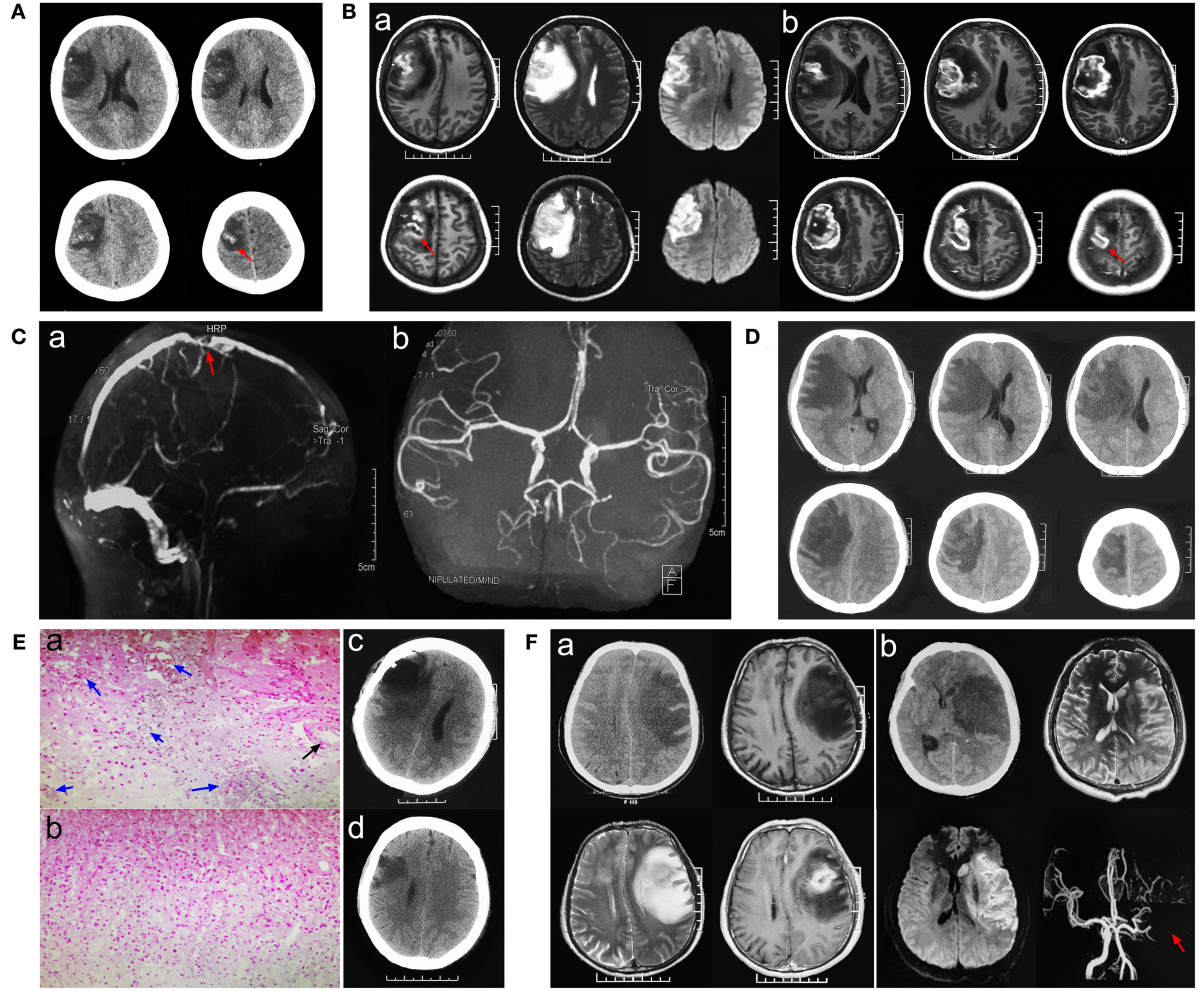
Pathological neuroimaging studies per case showed evidence of venous infarction secondary to cortical venous thrombosis (CVT). **(A)** Initial stage computed tomography (CT) showed hypodensity with some hyperdensity in the right frontal–parietal lobes. **(B**a**)** Magnetic resonance imaging (MRI) showed low-intensity signals mixed with high-intensity signals on T1-weighted images and high-intensity signals mixed with low-intensity signals on T2-weighted images in the right frontal–parietal lobes. **(B**b**)** Contrast-enhanced MRI revealed focal circular heterogeneous enhancement of the lesion. **(C**a**)** Magnetic resonance venography (MRV) showed cortical veins, and the corresponding parts of the superior sagittal sinus were sporadically visualized, which indicated CVI. **(C**b**)** Magnetic resonance angiography (MRA) showed that the main arteries and their branches were normal. **(D)** Progressive CT showed expanded edema and a significant shift in the midline. **(E**a,b**)** Histopathologic analysis revealed dilated small veins with congestion or thrombosis (blue arrow), while normal arterial structure and lumen (black arrow) and focal necrosis and inflammatory infiltration (green arrow) were found. **(E**c**)** CT showed that part of the necrotic tissue was removed 24 h post-operatively. **(E**d**)** Three-month follow-up post-operatively. **(F**a**)** A case of glioblastoma was confirmed by pathology. **(F**b**)** Imaging characters in a case of middle cerebral artery infarction.

A 20-year-old female patient was admitted to a hospital with complaints of headache for the past 4 days and numbness and weakness in her left limbs. The patient initially experienced headache and weakness and numbness in her left upper limb. Weakness and numbness in the left upper limb then progressed to the fingers and thumb of the left hand and gradually to the left lower limb. Neurological examination showed grade 1 muscle strength in the left upper limb, grade 3 muscle strength in the left lower limb, and hypoesthesia in the left limb. She had a cesarean section 5 days earlier. Non-enhanced computed tomography (NECT) revealed hypodense lesions, which enclosed hyperdense lesions, in the right frontal–parietal lobes (Figure 1A). So, could the initial diagnosis be acute cerebral infarction or glioma hemorrhage? Magnetic resonance imaging (MRI) showed low-intensity signals mixed with high-intensity signals on T1-weighted images and high-intensity signals mixed with low-intensity signals on T2-weighted images in the right frontal–parietal lobes. Hemorrhagic infarction was suspected, but glioma could not be excluded (Figure 1Ba). Laboratory tests showed that neuron-specific enolase (NSE) was 51.45 ng/ml (normal range: 0–16.3 ng/ml) and D-dimer was normal. Subsequently, magnetic resonance angiography (MRA) showed that the main arteries and their branches were normal; therefore, arterial cerebral infarction was excluded (Figure 1Cb). Magnetic resonance venography (MRV) showed cortical veins, and the corresponding parts of the superior sagittal sinus were sporadically visualized, which indicated CVI (Figure 1Ca). Furthermore, contrast-enhanced MRI revealed focal circular heterogeneous enhancement of the lesion, which suggested glioma hemorrhage, but cerebral infarction could not be ruled out (Figure 1Bb).

After symptomatic treatment, the patient's consciousness gradually deteriorated (awake-somnolence-lethargy), presenting grade 0 left limb muscle strength and loss of sensation. Emergency computed tomography (CT) showed expanded edema and a significant shift in the midline, possibly leading to the development of a hernia (Figure 1D). Because of the patient's increased consciousness and severe limb dysfunction, a craniotomy was performed to explore the lesion and, if necessary, a decompressive craniectomy was performed to relieve intracranial hypertension. Intraoperative findings were as follows: the lesion tissue was slightly higher than the bone window after cutting the dura mater; the brain tissue was dark gray; and the lesion boundary was relatively clear and located between the sulci. Intraoperative freezing reported no heterogeneous tumor tissue after the removal of the hemorrhagic and necrotic lesions. The brain tissue collapsed below the bone window, the dura mater was decompressed and sutured, and the bone flap was reset. Histopathological results were consistent with cerebral infarction: gliosis with inflammatory exudation, necrosis, vasodilation, and thrombosis (Figure 1Ea,b).

The final diagnosis was CVI. Post-operatively, necrotic tissue was removed (Figure 1Ec) the patient was administered low-molecular-weight heparin. One week later, the patient was conscious and had grade four left upper limb muscle strength, grade five left lower limb muscle strength, and decreased sensation. Rehabilitation treatment was provided after discharge. The left thumb showed numbness and slight weakness at the 3- (Figure 1Ed) and 6-month follow-ups, but no neurological symptoms were observed.

## Discussion

**Figure 2:**
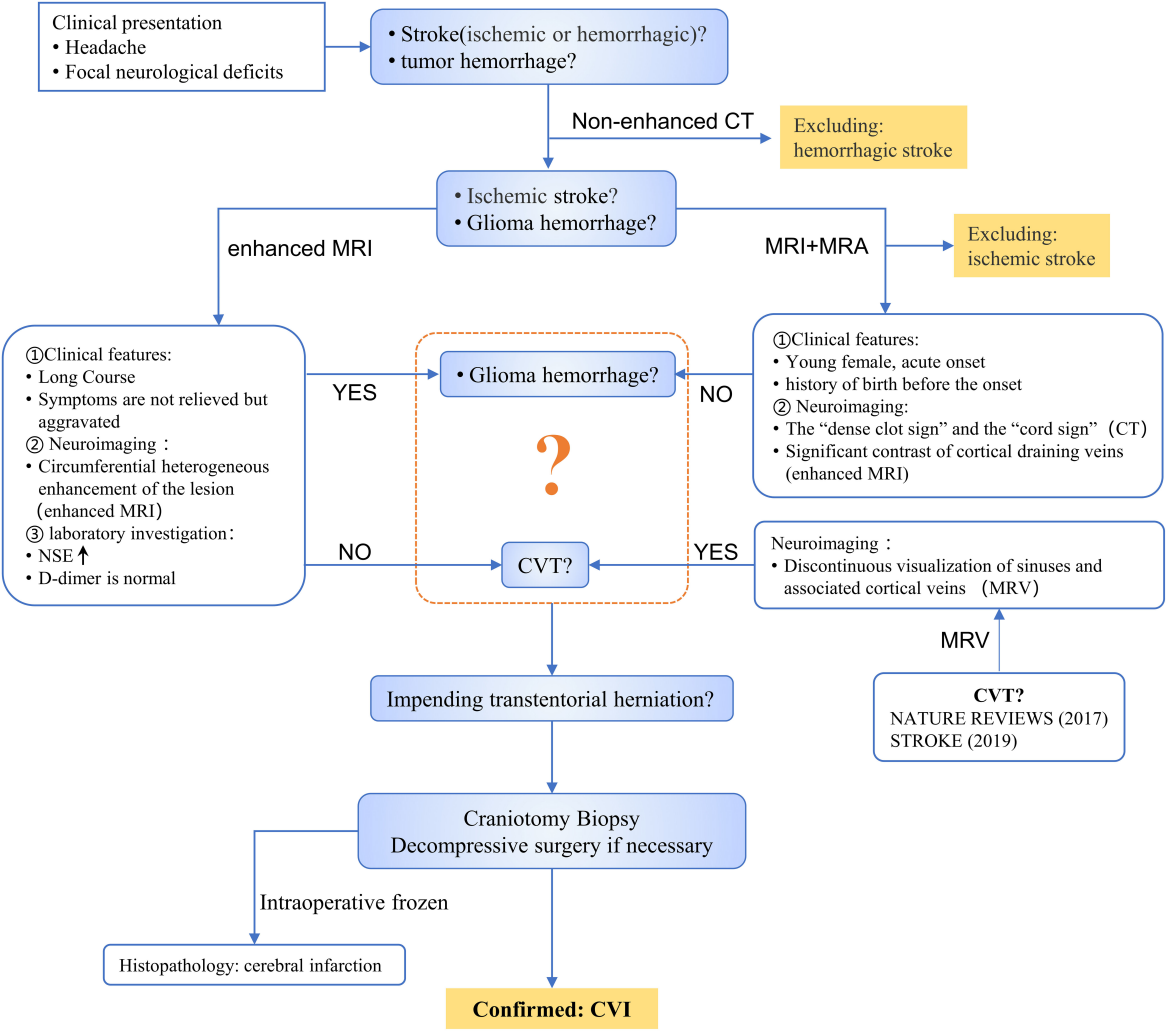
Differential diagnosis and treatment process of case.

This was a rare case of CVI secondary to cortical venous thrombosis (CVT), which was illusory and difficult to diagnose considering the presence of glioma hemorrhage. As a result, the diagnosis could not be confirmed until the pathological results of surgical exploration were available. The process used to diagnose this case is shown in Figure 2. Due to the atypical clinical and imaging manifestations of CVT, the condition is easily misdiagnosed as glioma (Figure 1Fa) or cerebral artery infarction (Figure 1Fb) (Yu et al., 2016). Detailed identification of CVI, arterial cerebral infarction, and glioma is shown in Table 1. CVI is most common in patients with CVT or venous sinus thrombosis (Coutinho et al., 2014) but can also be secondary to trauma (Harris et al., 2021) or iatrogenic cortical venous injury (Cai et al., 2021). Pregnant or postpartum women and young women taking contraceptives are considered high-risk groups and account for 75% of CVT cases (Farooqui et al., 2021). Acute or subacute onset mainly manifests as headache, focal neurological deficits, and seizures, among others. CVT is often associated with venous sinus thrombosis secondary to hemorrhage (Afifi et al., 2020). Imaging studies, such as CT and MRI, are crucial because they are used to observe the direct and indirect signs of CVT and to confirm diagnoses, including the “dense clot sign” and “cord sign,” as well as indirect signs, including lobar hemorrhage, subarachnoid hemorrhage, and focal cerebral edema. Cortical venous filling defects in MRV/CTV are helpful for the diagnosis of CVT (Dmytriw et al., 2018). Due to the large anatomical variability of cortical veins and the involvement of small veins, digital subtraction angiography (DSA) is not easy to detect and invasive DSA is not recommended (Song et al., 2021). If the nature of the lesion is still uncertain, biopsy of the lesion (intraoperative freezing) can be explored to confirm its diagnosis and guide its treatment.

**Table 1:** The detailed identification of glioma, arterial cerebral infarction, and cortical venous infarction.

	**Glioma**	**Arterial infarction**	**Venous infarction**
Age/sex	Middle and elderly/uncertain	Middle and elderly/male > female	Young/female > male
Pathogenesis	–	Arterial mural thrombosis	Draining vein or sinus thrombosis
Risk factors	Genetic mutation	Diabetes/hypertension/atrial fibrillation	Pregnancy/postpartum/oral contraceptives
Onset characteristics	Chronic onset, progressive worsening of symptoms	Acute onset, the disease peaks in about 5–7 days, and then gradually eases	Acute/subacute onset, the peak is delayed compared with arterial infarction
Clinical presentation	Chronic headache, neurological deficits, seizures	Acute headache, neurological deficits, seizures (no specificity)	Acute/subacute headache, neurological deficits, seizures (no specificity)
CT/MRI	• Mostly located in the subcortical white matter area, involving single or multiple lobes, with unclear borders and obvious peritumoral edema; • Annular inhomogeneous reinforcement. Scattered bleeding spots or hematoma formation within the lesion. (Figure 1Fa)	• Consistent with the blood supply range of the responsible artery, involves the cortex, subcortical, and deep brain; • Triangular or fan-shaped lesions with well-defined borders, and sulcus reinforcement; • Reperfusion hemorrhage after infarction (1/2 cases)	• Consistent with the area of cortical venous drainage, located in the cortex and sub-cortex, with marked brain swelling • Gyrus-like enhancement and obvious enhancement in the veins or venous sinuses at the site of thrombosis (Figure 1Bb red arrow). Bleeding in the acute phase (2/3 cases), direct sign: “dense clot and cord sign” (Figures 1A,Ba red arrow)
MRV/CTV	No abnormality in blood vessels	The responsible artery filling defect (Figure 1Fb red arrow)	The draining vein or sinus filling defect (Figure 1Ca red arrow)
Treatment	Surgery + chemoradiotherapy	Anticoagulation/endovascular therapy	Anticoagulant therapy
Prognosis	Short-Term effect is favorable	partial neurological deficit	Prompt treatment is favorable

In this case, due to CVI secondary to venous thrombosis, hemorrhage, and edema after infarction can cause a massive effect. When a hernia is about to occur, some of the hemorrhagic and necrotic lesions should be removed, and the dura should be decompressed and sutured to reduce intracranial pressure. CVI was ultimately confirmed and low-molecular-weight heparin was recommended in the acute phase (Silvis et al., 2017) with a good prognosis. Several studies (Ropper and Klein, 2021) showed a favorable outcome in patients with CVT and impending hernia who had undergone decompressive surgery.

## Data availability statement

The original contributions presented in the study are included in the article/supplementary material, further inquiries can be directed to the corresponding author.

## Ethics statement

The studies involving human participants were reviewed and approved by Tangdu Ethics Committee. The patients/participants provided their written informed consent to participate in this study. Written informed consent was obtained from the individual(s) for the publication of any potentially identifiable images or data included in this article.

## Author contributions

DF: conceptualization, methodology, and writing—original draft. LZ: writing—original draft. HQ: supervision. QC: writing—review and editing. All authors contributed to the article and approved the submitted version.
